# Multisource feedback in medical students’ workplace learning in primary health care

**DOI:** 10.1186/s12909-022-03468-7

**Published:** 2022-05-25

**Authors:** Karin Björklund, Terese Stenfors, Gunnar H. Nilsson, Charlotte Leanderson

**Affiliations:** 1 Department of Neurobiology, Care Sciences and Society, Karolinska Institutet, Stockholm, Sweden; 2 Department of Learning, Informatics, Management and Ethics, Karolinska Institutet, Stockholm, Sweden

**Keywords:** Communication and patient-centredness, Medical students, Multi-source feedback, MSF questionnaire, Workplace learning

## Abstract

**Background:**

In medical students’ workplace learning, feedback is important for effective learning regarding communication and clinical skills. The provision of multisource feedback (MSF) in clinical practice with focus on the patient’s perspective is rarely addressed in the literature. The overall objective was to explore the experience of MSF in medical students’ clinical learning in primary healthcare (PHC).

**Methods:**

In the study, patients provided feedback by use of the Patient Feedback in Clinical Practice (PFCP) questionnaire. By use of adapted PFCP questionnaire versions peers and clinical supervisors provided feedback and students performed a self-evaluation. The MSF learning activity was evaluated using surveys (4-point Likert scale/open-ended questions), (students (*n* = 26), peers (*n* = 9) and clinical supervisors (*n* = 7)). Data were analysed using descriptive and qualitative content analysis.

**Results:**

Results (mean 4-point Likert scale) from participants evaluation of the MSF learning activity visualises the value of feedback in terms of patient-centred communication (students 3.50, peers 2.44 and clinical supervisors 3.57), guidance for further training (students 3.14, peers 2.89 and clinical supervisors 3.00) and clarification of pedagogical assignment (students 3.14, peers 2.89 and clinical supervisors 3.00). Thematic analysis of participants’ free-text answers in the evaluation surveys resulted in three themes: (1) applicability of the MSF, (2) MSF – collaborative learning process and (3) MSF as a facilitator in students’ clinical skills development. The participants experienced that the written MSF provided multi-facetted perspectives, which contributed to students’ and peers’ clinical and communication learning. MSF experience also enhanced clinical supervisors’ feedback regarding communication skills, targeting the supervisors’ pedagogical assignment.

**Conclusion:**

Our findings indicate that MSF provided directly after a patient encounter, using the PFCP questionnaire as feedback provider, could be an adequate learning activity for medical students’ workplace learning. The MSF, provided through the PFCP questionnaire, was experienced to neutralise and operationalise the provision of concrete feedback, facilitating peers’ learning and clinical supervisors’ tuition. The results visualise the importance of patients in MSF, as a valuable resource in students’ workplace learning. Our study implies that this learning activity could be an applicable tool to facilitate learning and pedagogic development in clinical education in PHC.

**Supplementary Information:**

The online version contains supplementary material available at 10.1186/s12909-022-03468-7.

## Background

In workplace learning, medical students are provided with real-life situations in which the students can develop and attain workplace competencies [1]. The students can, through interactions with patients, train clinical competencies facilitated by the clinical supervisors and in addition further developed in learning with peers [2].

Research emphasises feedback on performance during a patient encounter as central for students’ learning [3–6]. Especially when feedback is provided in relation to a specific task or process [6, 7], for example, provided immediately after a patient encounter [8].

During clinical rotations, the primary source of feedback is usually the clinical supervisor [3, 4, 9], assessing the students’ performance during a patient encounter [3, 10]. Additionally, students sometimes receive feedback from peers and sometimes perform a self-evaluation [3, 4, 9]. Feedback from peers has been described as facilitating students’ clinical skills learning and can provide a guide for future decisions and plans for further improvement [11, 12]. However, peer evaluations depend on trust that requires attention to the confidentiality in between students [5, 7, 13, 14]. Peer feedback can be undermining, divisive, and destructive if the skills and knowledge domains used for peer feedback are not appropriately adhered to the adequate level of competence [5, 7, 14] and administered in a safe learning environment [14]. Students’ self-reflective learning process can be stimulated through self-evaluations of own clinical performance and thereby induce the identification of gaps in knowledge and skills and areas for further clinical training [4, 9, 15]. Patients more seldom provide feedback to medical students about the patients’ subjective experiences of the student’s ability to communicate and apply patient-centeredness during a student-led encounter [3, 10, 16]. However, previous research shows that patients’ feedback is a valuable addition in students’ clinical learning [4].

Receiving multi-source feedback (MSF), including feedback from patients, peers and clinical supervisors, sometimes in combination with students’ self-evaluation, can play an important role in students’ clinical training [4, 16–18], hence illustrating required levels of clinical skills and facilitating self-reflection [19, 20]. However, students seldom receive MSF during their clinical rotation because of numerous barriers attributed mainly to logistical and organisational perspectives [17].

Only a few studies were found where different groups of participants used the same feedback questionnaire or evaluation form to provide feedback to a medical student about a specific encounter [21, 22]. Previous studies in which medical students receive MSF are usually based on different questionnaires and evaluation forms embedded in different teaching and learning programmes [4, 19, 23, 24]. This means that the feedback provided is usually anonymous, delayed and provided after numerous patient encounters [4, 17]. In addition, the MSF is often obtained through assessment of a competence, illustrating overall performance, rather than a specific task. The MSF, provided through the PFCP questionnaire, was experienced to neutralise and operationalise the provision of concrete feedback, facilitating peers’ learning.

To provide MSF as a source for students self-directed learning [25, 26], pre-defined specific items addressing various patients’ perspectives of a student-led encounter could be used. Furthermore, it would also be valuable to explore peers’ and clinical supervisors’ experiences of participation in MSF.

The overall objective of this study was to explore the experience of MSF in medical students’ clinical learning in PHC, and we explore this through the following questions:

1. How do students experience to receive feedback from patients, peers and clinical supervisors and perform self-evaluation through a feedback questionnaire?
2. How do peers and clinical supervisors experience to provide feedback through a questionnaire?
3. Can written MSF adjacent to a patient encounter in PHC be a feasible learning activity during clinical rotations in PHC?

## Methods

### Study design

This study was conducted during a two-year period at the medical programme at Karolinska Institutet (KI), Sweden, and data were collected during the students’ workplace learning at six PHC centres, located in areas with varying socioeconomic characteristics in Region Stockholm. A mixed-methods research design was applied using an evaluation survey (4-point Likert scale and open-ended questions) to explore the students, peers, and clinical supervisors’ experiences of a multi-source feedback (MSF) learning activity [27]. The study comprised two steps: the provision of feedback in an MSF learning activity and evaluation of the participating in the MSF learning activity. Social constructivism was used as a conceptual framework in the design of this study and the MSF learning activity [28, 29].

### Participants

The participants in the study were medical students, the students’ clinical supervisors at PHC centres and the patients who were seen by the students.

### Inclusion criteria

Medical students at semesters 2–7, 9 and 11 with clinical rotation in PHC. Clinical supervisors in PHC centres. Patient’s age > 18 years, without a diagnosis of dementia, cognitive disabilities and/or mental disorders and who had agreed to participate in a student-led encounter at a PHC centre.

### Consent for the current study and ethical approval

To receive informed consent for the current study, e-mails were sent to the heads of the PHC centres, who had two or more students from semesters 2–7, 9 and 11 per clinical rotation. Six PHC centres approved participation. At those units, the students and clinical supervisors were invited to participate in the study by e-mail and participation was also offered on-site at the PHC centres. In addition, the ability for patients to participate in study was also offered on-site at PHC centres. All participants were informed through both oral and written information, and written consent was obtained from all participants. Material for data collection and protocol for storing and utilising the data were approved by the Regional Ethical Review Board in Stockholm (Dno: EPN 2017–1574-31–1).

## Context

In medical programme, workplace learning is performed in PHC centres during nine of the11 semesters. Clinical rotations usually last between four and seven days per semester, with one to four students at each PHC centre. The students progressively train communication and patient-centredness during their workplace learning in PHC [30–33], which is taught in alignment with the generic model for doctor-patient communication at Maastricht Medical School [34]. The students perform different parts of the patient encounter in alignment with the learning objectives for the respective semester under direct and/or indirect supervision. To train clinical and communication skills, the students at the PHC centres predominantly meet patients with different problems or diseases that target the educational domains for each semester. Clinical supervisors often manage their own outpatient clinical work in parallel with their clinical supervisor assignment, tutoring one to four students.

### The PFCP questionnaire

The Patient Feedback in Clinical Practice (PFCP) questionnaire [35] was used to provide written multi-source feedback in the present study. The PFCP questionnaire is a newly validated and pilot tested questionnaire developed for patients’ written feedback to medical students in PHC [35]. The PFCP questionnaire was linguistically adapted for multi-source feedback and students’ self-evaluation. The content of the PFCP questionnaire is in alignment with the teaching of communication and patient-centredness in Swedish medical education [31–34] and based on generic models of communication and patient-centredness, for example, the Maastricht Medical School [34] and Calgary Cambridge Guides [31]. The PFCP questionnaire comprises 19 items to evaluate patient perspectives of student’s performance during an encounter by use of a 4-point Likert scale with clarifying text for each scale step (from strongly disagree to strongly agree and including ‘not applicable’ and ‘performed by supervisor’). After each item and at the end of the questionnaire, there is space for free-text comments. To further explore patients’ experiences of the student-led encounter, three open-ended questions were added to the PFCP questionnaire.

### Adaptation of the PFCP questionnaire

In this present study, the PFCP questionnaire was adapted for peers’ and clinical supervisors’ feedback and for students’ self-evaluation with the intention to provide feedback regarding the student’s performed encounter in relation to domains in the PFCP questionnaire. The adaptation process included interviews with ten medical students from semesters 2, 7, 9 and 11 and three clinical supervisors at five PHC centres. The medical students and clinical supervisors who participated in adaptive phase regading the questionnaire did not participate in the MSF learning activity in the present study. The adaptation process was consolidated through iterated discussions within the authors’ team. All items were linguistically adjusted to obtain an objective assessment, hence focusing on the patient perspective of patient-centredness and communication skills from the respective participants’ group point of view. The adaptation process resulted in three versions of PFCP questionnaires, exploring peers’, clinical supervisors’ and students’ own experience of the encounter with a focus on a patient perspective, presented in Table 1.

**Table 1:** The Patient’s Feedback in Clinical Practice (PFCP) questionnaire, inclusive three added patient questions (12, 21 and 22) (in roman) and the adapted version for students, peers and clinical supervisors’ version (in italics)

1	Did you have the opportunity to explain the reason for your visit or what had happened since you last visited the doctor? *From your perspective: Did the patient have the opportunity to explain the cause of concern or what had happened since the patients last visited the doctor*?
2	Did you have the opportunity to explain your own thoughts regarding your problems? *From your perspective: Did the patient have the opportunity to explain his/her own thoughts regarding the cause of concern?*
3	Did you have the opportunity to explain if there was something that worried you regarding your problems? *From your perspective: Did the patient have the opportunity to explain if there was something that worried her/him regarding the cause of concern?*
4	Did you have the opportunity to express if there was something specific you wanted to be performed/initiated during the consultation? *From your perspective: Did the patient have the opportunity to express if there was something specific he/she wanted the doctor to perform/initiate during the consultation?*
5	Did the student confirm with you that he/she understood your cause of concern correctly by summarising what you told him/her? *From your perspective: Did the student/you confirm with the patient that the student/you understood the cause of concern correctly by summarizing what the patient told the student/you?*
6	Did the student explain his/her medical questions, so you understood why they were asked? *From your perspective: Did the student/you explain the medical questions, so the patient understood why they were asked?*
7	During the clinical examination, did the student explain why certain examinations were performed? *From your perspective: During the clinical examination, did the student/you explain why certain examinations were performed?*
8	Did the student take into consideration your own thoughts regarding your problem when you discussed the follow-up plan/treatment? *From your perspective: Did the student/you take into consideration the patient's own thoughts regarding the patient's problem when the student/you discussed the follow-up plan/treatment?*
9	Did you receive information/explanation from the student which made it possible for you to participate in the planning of care/treatment? *From your perspective: Did the patient receive information/explanation, which made it possible for the patient to participate in decisions regarding the patient’s own care/treatment?*
10	Did the student provide information about suggested care/treatment in a way that you understood? *From your perspective: Did the student/you provide information about suggested care/treatment in a way that the patient understood?*
11	Did the student provide information about medication in a way that you understood? *From your perspective: Did the student/you provide information about medication in a way that the patient understood?*
12—Patient	Did you percieve that you received enough information regarding your eventual medication?
13	Did the student provide information in a way that you understood regarding symptoms that call for immediate contact with healthcare? *From your perspective: Did the student/you provide information in a way that the patient understood regarding symptoms that call for immediate contact with healthcare?*
14	Did the student ask if the information you were given was interpretable? *From your perspective: Did the student/you ask the patient if the information the student/you gave was interpretable?*
15	Did you have the opportunity to bring up questions you had before the visit regarding your cause of concern? *From your perspective: Did the patient have the opportunity to bring up questions that the patient had before the visit regarding the cause of concern?*
16	Did the student involve you in the decision-making process regarding your care/treatment? *From your perspective: Was the patient involved in the decision-making process regarding the patient’s own care/treatment?*
17	Were you involved in the decision-making process regarding your care/treatment to the extent you wanted? *From your perspective: Was the patient involved in the decision-making process regarding the patient care/treatment to the extent the patient wanted?*
18—Patient	Are you satisfied with the initial plan that was decided upon together with the student?
19	Did you experience that the student treated you with compassion and consideration? *From your perspective: Was the patient treated with compassion and consideration?*
20	Did you experience that the student treated you with respect and dignity? *From your perspective: Was the patient treated with respect and dignity?*
21—Patient	Based on the information you received during your visit, will you follow the recommended treatment plan, such as following prescription?
22—Patient	In light of your visit today, do you feel that you are in the need of additional consultation due to your current symptom/problem?

### Data collection

After participation in the MSF learning activity students, peers and clinical supervisors evaluated their experience by filling in an evaluation survey (4-point Likert scale and open-ended questions). The data was collected by one of the authors (KB).

### Evaluation survey

Prior to data collection, in order to explore study participants’ experiences of MSF, an evaluation survey, with adapted versions for students, peers and clinical supervisors, was developed through iterative discussions between the authors. The evaluation surveys included questions with a 4-point Likert scale and free-text answers, presented in Table 2. The patients’ experiences of providing feedback to the medical students through the PFCP questionnaire has been explored in a previous study [35].

**Table 2:** The students’, peers’ and clinical supervisors’ evaluation surveys

	**Student self-evaluation**	**Peer evaluation**	**Clinical supervisor evaluation**
1	How was your experience of receiving written feedback?	How was your experience evaluating your peer’s performance during the patient encounter?	How was your experience of providing written feedback?
2	How did you experience evaluating your performance of the patient encounter?		How was your experience using the feedback in your clinical tuition?
3	Did your self-evaluation differ in relation to the other participants’ feedback? Yes/No If yes, in what way?		Did your feedback differ in relation to the student self-evaluation and to the other participants’ feedback? Yes/No If yes, in what way?
4	The multi-source feedback provided valuable information regarding my ability to apply patient-centred communication.1– 4 + not relevant	Providing feedback to peer helped me to further clarify the importance of patient-centred communication.1– 4 + not relevant	The multi-source feedback and the student’s self-evaluation provided valuable information regarding the student’s ability to apply patient-centred communication.1– 4 + not relevant
5	Please give examples of how the multi-source feedback clarified your ability to apply patient-centred communication.	Regarding question four, please give examples in what way?	Please exemplify how the multi-source feedback clarified the student’s ability to apply patient-centred communication.
6	The multi-source feedback provided guidance for future training of clinical skills. 1– 4 + not relevant	Providing feedback to peer gave me guidance regarding how I could train and develop clinical skills.1– 4 + not relevant	The multi-source feedback and student’s self-evaluation provided guidance for the student’s future training and development of clinical skills. 1– 4 + not relevant
7	Please give examples of how the multi-source feedback facilitated your future training regarding clinical skills.	Regarding question six, please give examples in what way?	Please exemplify how the multi-source feedback facilitated the identification of student's need for future training regarding clinical skills.
8	The multi-source feedback helped to visualise my pedagogical assignment during the dialogue with the patient. 1– 4 + not relevant	To provide feedback helped me to visualise the pedagogical assignment during the dialogue with the patient.1– 4 + not relevant	The multi-source feedback and student’s self-evaluation helped to visualise the student’s pedagogical assignment during the dialogue with the patient.1– 4 + not relevant
9	Please give examples of how the multi-source feedback clarified your pedagogical assignment in the dialogue with the patient.	Regarding question eight, please give examples in what way?	Please exemplify how the multi-source feedback clarified the student’s pedagogical assignment in the dialogue with the patient.
10	Describe the major outcome from performing self-evaluation and receiving feedback.	Describe the major outcome of providing feedback.	Describe the major outcome of giving and using the feedback in your pedagogical assignment as a teacher.
11	Please add if you have further comments.	Please add if you have further comments.	Please add if you have further comments.

### Setting and procedure

- During student-led patient encounters, the peer’s and/or the student’s clinical supervisor participated. Adjacent to the encounter the patients filled in the PFCP questionnaire in the waiting room. Peers and clinical supervisors individually filled in respective versions of the adapted PFCP questionnaire to provide feedback for the student. Students also performed a self-evaluation by filling in their version of the adapted PFCP questionnaire. The participants were not anonymous in provision of feedback in the PFCP questionnaire. In the study the data is anonymised. The participants’ scoring of items in the PFCP questionnaires and examples of free-text comments will not be further discussed in this paper but are found in Additional files, [see Additional files 1 and 2].
- The student and clinical supervisor took thereafter part in the MSF in a feedback session following the patient encounter.
- Peers did not participate in the feedback sessions.
- Students, peers and clinical supervisors evaluated their use of use of the PFCP questionnaire by filling a respective version of an evaluation survey. The evaluation survey targeted the participants experience of MSF learning activity, including providing and receiving written feedback by use of the PFCP questionnaire. Some participants took part in multiple MSF sessions, although each participant completed an evaluation of the MSF learning activity only once. The participants were anonymous while filling in the respective version of the evaluation survey.
- The PFCP questionnaire forms and the evaluation surveys were collected, and all the data were documented in an Excel spreadsheet by KB. The participants’ scoring of items in the PFCP questionnaires and examples of free-text comments are presented in Additional files [see Additional files 1 and 2].

### Data analysis

#### Evaluation of MSF participation – Quantitative data

Data from the students’, peers and clinical supervisors’ evaluation surveys of MSF participation (questions with a 4-point Likert scale) were analysed using descriptive statistical methods using SPSS Statistics 26 software (IBM, Armonk, NY). The mean, standard deviation (SD) and range for each question were calculated.

#### Evaluation of MSF participation – Qualitative data

The qualitative data from students’, peers’ and clinical supervisors’ free-text comments from the evaluation surveys were analysed using qualitative content analysis [36]. The qualitative analysis was performed separately for each group of participants according to the following process:

To obtain an overview of the participants’ experiences of receiving and providing feedback, KB and CL repeatedly read all the text from the evaluation surveys. Meaning units were identified, condensed with perceived key areas of content, compared to ensure consistency and sorted into categories. The underlying meanings of the categories and notes from the first impression were merged and interpreted. The categories from the different group analyses were interpreted and merged, resulting in three themes. The themes were discussed and established by the authors’ team.

## Results

### Participants

Information about the participants in the MSF setting and the participants evaluation regarding experiences of the MSF learning activity is presented in Table 3 and 4.

**Table 3:** Number of participants in the MSF setting

Number of participants	Number	Age in range	Semester
Patients	43	18—91	
Peers	16	18—38	5 and 7
Clinical supervisors	12	25—66	
Medical students	33	18—38	5, 7 and 11

**Table 4:** Number of participants evaluating the MSF learning activity

Number of participants	Number	Age in range	Semester
Medical students	26	18—38	5, 7 and 11
Peers	9	18—38	5 and 7
Clinical supervisors	7	25—66	

### Experience of the MSF

The analysis of the students’, peers’ and clinical supervisors’ experiences of MSF feedback, here based on the qualitative data derived from free-text answers in the evaluation surveys, resulted in three themes: 1) applicability of MSF, 2) MSF—collaborative learning process and 3) MSF as a facilitator in students’ clinical skills development. Table 5 presents the mean, standard deviations and range for questions 4, 6 and 8 on a 4-point Likert scale from the students’, peers’ and clinical supervisors’ evaluation surveys. Table 6 present an overview of the content analysis and quotations from the free-text answers in the evaluation surveys about students’, peers’ and clinical supervisors’ experience of use of the adapted PFCP questionnaires for MSF.

**Table 5:** The students, peers, and clinical supervisors’ mean, SD and range values for questions with 4-point Likert scale in the evaluation surveys, visualising the value of the MSF in the field of patient-centred communication (question 4), guidance for further training (question 6), and clarification of pedagogical assignment (question 8)

	**Students**	**Peers**	**Clinical supervisors**
Question 4	3.50, 0.67, 1 – 4	2.44, 1.33, 1 – 4	3.57, 0.53, 1 – 4
Question 6	3.14, 0.99, 1 – 4	2.89, 1.67, 1 – 4	3.00, 0.58, 1 – 4
Question 8	3.14, 0.60, 1 – 4	2.89, 0.97, 1 – 4	3.00, 1.00, 2 – 4

**Table 6:** Overview of the content analysis and quotations from the free-text answers in the evaluation surveys about students’, peers’ and clinical supervisors’ experience of use of the adapted PFCP questionnaires for MSF

Theme	Subthemes	Quotations
Applicability of PFCP MSF	•The MSF setting	‘‘*Very good to receive this feedback*’. (Student) ‘*Good, nothing strange’*. (Peer)
	•The PFCP questionnaire usability for MSF	*‘Good tool for self-evaluation*’. (Student) ‘*Written, structured feedback is a great help and complement in my assessment of students*’. (Clinical supervisor)
MSF—collaborative learning process	•MSF as a facilitator for students’ and peers’ self-reflection	‘*Developing for both of us [student and peer]’*. (Student) *‘Self-critical thinking... was strengthened*’. (Peer)
	•MSF as a multi-perspective reinforcement in clinical learning	‘.*.. showed the patient’s perception... otherwise... difficult to understand...’* (Student) '*Gave me ideas of how I can conduct a patient encounter*’. (Peer)
MSF as a facilitator in the students’ clinical skills development	•MSF acknowledging students’ clinical performance	‘*Gave me increased self-confidence... easier to focus and maintain the “red thread”’*. (Student)
	•MSF as a motivator for further clinical training	*‘Ask about concerns, expectations, own thoughts... by asking adequate follow-up questions and to give more space’.* (Student) ‘*... clarifying... I need to work more on... being pedagogical (at the end of the consultation)... generally I have... good communication skills... [I] need to work harder to explain why I perform some examinations and include the patient in the treatment plan*’*.* (Student)

### Applicability of MSF

The theme applicability of MSF included two subthemes: 1) the MSF setting and 2) the PFCP questionnaire usability for MSF. The theme visualises the students’, peers’ and clinical supervisors’ experiences and perspectives of the MSF during a mutual patient encounter, as well as the questionnaire’s ability to provide adequate MSF to facilitate clinical learning.

### The MSF setting

The students, peers, and clinical supervisors s found the MSF setting during the patient encounter applicable for providing and receiving feedback by use of the adapted PFCP questionnaire. A few participants stated that they wanted more time to be allocated for the MSF activity, so they initially hesitated to participate. However, once they decided to participate, the entire cohort was content with the activity.

‘... more time to participate in settings like this would have been beneficial’. (Student).

‘... to provide feedback to each other is a good thing’. (Peer).

‘Good... important... as students often... unsecure of their performance’. (Clinical supervisor).

‘Important for the student’s development’. (Clinical supervisor).

The students and peers experienced the MSF learning activity as an allowing setting in which they experienced it comfortable to participate in and to provide and to receive feedback using the PFCP questionnaires. A few students initially expressed some anxiety over being assessed in an MFS setting. However, after having performed the encounter, all students were content with the setting.

‘Initially nervous... (what if you are really bad)... the aim is good’. (Student).

‘It went well, usually difficult to evaluate certain things you do routinely’. (Student).

‘... good to have to evaluate each other… if we had not been so comfortable with each other, we would never have pulled it off without these forms’. (Peer).

### The PFCP questionnaire usability for MSF

The students, peers and clinical supervisors experienced that the adapted PFCP questionnaire was an adequate feedback and self-evaluation tool that chronologically explored whether the participants perceived that the patient’s perspective was considered in important aspects and components of a patient encounter. The clinical supervisors stated that the structure and content of the questionnaire facilitated the incorporation of patients’ perspectives into the provided feedback. Only a few students and clinical supervisors experienced that they initially required some practice in filling in the PFCP questionnaire.

‘The feedback addressed all essential parts and components of a patient-centred encounter’. (Student).

‘... not overly difficult... just required some thinking to get acquainted with’. (Student).

‘... facilitates... the ability to provide good structured and constructive feedback to students... the questions requested... this... provides a basis... for the discussion regarding the content of the encounter’. (Clinical supervisor).

‘Written, structured feedback is a great help and complement in my assessment of students’. (Clinical supervisor).

In some cases, all the participants seemed to have the perceived understanding that some, or all aspects of patient-centredness were not applicable or necessary to apply during the encounter (e.g. patients with a serious disease, a difficult situation or a very short visit with a specific pre-defined reason, such as excision of a naevus).

‘Difficult... to apply on my consultation, the patient had a preunderstanding exactly of what was going to be discussed... it was a recurrence of disease’. (Student).

‘Uncomplicated visit with a vital patient’. (Clinical supervisor).

### MSF – Collaborative learning process

The theme MSF – collaborative learning process included two subthemes: (1) MSF as a facilitator for self-reflection and (2) MSF as a multi-perspective reinforcement in clinical learning. The theme includes aspects of how the inclusion of the MSF for students, peers and clinical supervisors was perceived to facilitate the addition of valuable perspectives for self-reflection.

### MSF as a facilitator for students’ and peers’ self-reflection

The MSF learning activity initiated a self-reflective learning process in students and peers, in which the items’ content facilitated awareness of a broader conceptual understanding of clinical performance.

‘... have to think by yourself about what you could have done differently’. (Student).

‘... see things in a way that you do not reflect on when you perform the patient encounter by yourself’. (Peer).

### MSF as a multi-perspective reinforcement in clinical learning

The students, peers and clinical supervisors experienced that the different participants scored the students’ performances during the encounter slightly differently. The students tended to rate their own performance lower compared to the clinical supervisors and patients. Patients evaluated the students’ performance as higher, in alignment with the clinical supervisors. The variation in estimations initiated a reflective discourse.

‘My self-evaluation was harsher than... the one my peer performed. The patient was more satisfied than I was’. (Student).

‘My feedback was in alignment with the patient’s, the student underestimated own performance constantly’. (Clinical supervisor).

Participation in the MSF gave multifaceted perspectives of learning and teaching adjacent to a patient encounter.

‘The feedback from the supervisor gave a picture of how my consultation was experienced by another person in the room’. (Student).

‘Interesting... I must familiarise myself with the patient’s situation... see it from their perspective’. (Student).

‘ Gain insight into what they [student and patient] come up with in real time... good... to see how others work out information for the patient… easy to miss something... only visible when you see it from the observer perspective... to be meticulous with the information exchange...’. (Peer).

‘When the patient can participate and express thoughts through feedback... clearer for the student how communication functions’. (Clinical supervisor).

As observers of the student-led patient encounter and providers of feedback through the adapted PFCP questionnaire, the peers described that they received valuable perspectives about how a patient encounter, including patient-centred techniques, could be conducted. Through this information, the peers also experienced that they were acknowledged in their own clinical competencies and facilitated in their own clinical learning progression.

‘Ask clearly about the patient’s ideas, concerns and expectations... some medical terms the patient might not understand... learn for myself... consider if it is something that [I] have missed’. (Peer).

The patient-focused feedback enhanced the patients’ agenda and patient-centredness throughout the encounter, thereby providing valuable perspectives to include in the clinical supervisors’ provision of feedback to the students.

‘Explanation to the patient why you perform the clinical examination... explain more why I perform different examinations... helps the student to think about it’. (Clinical supervisor).

‘For instance, that the student missed summarising regarding the patient’s ideas, concerns and expectations’. (Clinical supervisor).

### MSF as a facilitator in students’ clinical skills development

The theme MSF as a facilitator in students’ clinical skills development included two subthemes: 1) MSF acknowledging students’ clinical performance and 2) MSF as a motivator for further clinical training.

### MSF acknowledging students’ clinical performance

The students experienced that the MSF added a contextualised acknowledgement of their performance during the encounter, which provided increased self-confidence in clinical practice.

‘Strengthened my self-esteem. Made me... more satisfied with my performance’. (Student).

### MSF as a motivator for further clinical training

The students described that relating MSF to their own self-evaluation helped them to visualise and underpin the interpretation of their own performance in clinical practice and how to improve their clinical and communication techniques. Furthermore, the students described that the MSF functioned as a reminder of the applicability and importance of patient-centred communication as a working method throughout a patient encounter.

‘... [I] can be clearer while summarizing the patient’s ICE [ideas, concerns and expectations]... some issues may be more sensitive for some patients than for others... I should maintain working with patient-centred consultation’. (Student).

‘The importance of ICE [ideas, concerns and expectations]’. (Student).

‘Obtain the patient’s ideas, concerns and expectations... make own work as clinician easier... understand the patient’s agenda... confirm... the patient appreciates patient-centred consultation’. (Student).

The students also stated that their medical and pedagogical assignment as a health provider was enhanced by use of the MSF, hence addressing the necessity of not only being able to explain and communicate information, but also to theoretically master the discussed subject or, for example, the performed clinical examination.

‘e.g. to inform the patients why I ask certain questions... [I] need to be better at informing the patients why I perform certain examinations’. (Student).

‘[I] need to know what kind of examination I perform during the clinical examination and specific tests and not only to know what to look for in order to provide an adequate explanation for the patient.’ (Student).

## Discussion

To provide additional feedback to medical students, a multi-source feedback (MSF) learning activity was developed. The learning activity focused on students’ clinical training in communication and patient-centeredness during workplace learning. The results show that the students, peers and clinical supervisors experienced that the written MSF provided a multifaceted patient-focused perspective of learning and teaching. The students described how the MSF acknowledged their clinical performance, facilitating the identification of areas for improvement regarding clinical and communication skills, hence facilitating the students’ further clinical training. The peers stated that they received additional information, which facilitated their own progression in clinical learning. Furthermore, the clinical supervisors experienced that the patient-focused feedback provided valuable perspectives to include in feedback provided to the students.

Previous research of MSF describes challenges to overcome such as logistical, organisational and educational relationships and time [17, 20, 37, 38]. One important aspect of the implementation of MSF as a learning activity is that clinical supervisors, students and peers should experience the learning activity as beneficial and motivational as a tool for clinical learning [17]. In the current study, clinical supervisors, peers and students experienced the MSF provision as beneficial and motivational, which also prompted an increased interest in future participation in MSF learning activities, which is in alignment with previous research [17]. Furthermore, the results indicate that the MSF adjacent to a patient encounter could be a feasible learning activity in primary healthcare.

The participants stated that the PFCP questionnaire provided a structure for MSF. This is consistent with theories of concrete, interpretable and actionable feedback [5, 7, 39, 40]. The MSF, as operationalised through the questionnaire, was perceived as a valuable non-competitive contribution to the students’ and peers’ self-directed learning processes.

To further facilitate students’ self-directed learning process, it is of importance that students can relate and interpret information provided in the written MSF [25, 26]. Therefore, the participants’ feedback was provided adjacent to the patient encounter. Previous research has emphasised the importance of anonymous feedback as a favourable approach to creating a safe learning environment for providing feedback and managing relationship dependencies [41, 42]. Despite the MSF not being provided anonymously, the students and peers described that the MSF learning activity and feedback, promoted a transparent and non-judgemental framework for evaluation. Due to the enhanced transparency of the evaluated items, the MSF learning activity, reduced relationship barriers between the participants and was perceived to create an open learning environment. These findings suggest that non-anonymous feedback through a questionnaire with pre-defined specific content for evaluation could be included in students' learning.

In our study, some students tended to underestimate their own performance, which is alignment with previous studies [4, 43]. The patients’ scoring in the PFCP questionnaire were generally higher than the students’ own scoring, which has also been found in previous studies [4, 23]. In general, the clinical supervisors’ scores were also often slightly higher than the students’ own scoring, which has been described in previous studies as well [4]. Research has shown that students have to consider and reflect on the accuracy of the different ratings of items and the value of the provided information as a tool in their learning process [13]. In the present study, the MSF was believed to address the gap between the ideal and current reality. Additionally, the students expressed that the slight variation in the participants’ scoring facilitated a self-reflective learning process with increased acknowledgement of their own level of performance during the encounter, which is in alignment with previous work [20, 44].

Our results are in alignment with theories of self-directed learning, which previous research has emphasized as an important tool for facilitating students’ professional learning [6, 45]. The MSF learning activity, including written feedback, could thereby be a valuable complement to students’ and peers’ clinical training during workplace learning in PHC. The results of the MSF learning activity indicate that the design is in alignment with self-directed learning within a social constructivist framework [46, 47].

Our results are in line with Burgess et al.’s studies [13, 40] regarding peer feedback, showing that by filling in the PFCP questionnaire and providing feedback, the peers were driven to analyse and reflect on their own knowledge and skills, through an initiated self-reflective process. Furthermore, by observing and providing feedback, the peers gained in-depth knowledge, which has also been seen in previous studies [13, 39]. Through the multifaceted perspectives, the peers, as well as the students, experienced that they in order to apply patient-centredness needed adequate and solid theoretical medical knowledge.

In addition to the peers, the clinical supervisors described that participating in the MSF learning activity, facilitated the clinical supervisors’ pedagogical assignment. The clinical supervisors also experienced that they had received a multifaceted additive perspective of patient-centredness regarding the student’s performance during the encounter to consider and include in discussions with the student. Previous research has described the necessity of an increased awareness of the process and structure during an encounter to facilitate students’ ability to identify areas for development [48]. However, further studies are required to explore these aspects.

In our study, participants had the perceived understanding that all aspects of patient-centredness were not applicable or necessary to apply during all encounters. This was not explored further in the current study, but it is in alignment with previous work [49] and should be further studied within the context of patient-centredness and medical education.

### Strengths and limitations

A strength of the present study was that both qualitative and quantitative data were included, representing multiple perspectives. Furthermore, it included students from several semesters at six PHC centres representing diverse socioeconomic populations, which is known to increase the credibility and dependability of a study [50]. Another strength was that the participants could use a questionnaire with same content and structure, which experienced to capture the students’ ability to communicate and apply patient-centredness. The questionnaire was composed in alignment with intended learning outcomes for the Swedish medical education context and follows the generic model from work at Maastricht Medical School [34] and Calgary Cambridge Guides [31], strengthening the authenticity of the feedback. There are, though, some limitations in our study that should be considered. First, the number of participants in each MSF learning activity differed, and there were only a few MFS settings where full range of participants participated and provided feedback. However, the results indicate that the number of participants providing feedback was inferior in this setting as use of the PFCP questionnaire set the state regarding the feedback content and structure, enabling addition of layers of transparent feedback facilitating students clinical learning. Another limitation is that the patients’ experiences of participating in the MSF learning activity and provision of feedback by use of the PFCP questionnaire are lacking. However, the patients’ perspectives of the PFCP questionnaire for feedback provision have been explored in a previous study [35], indicating that the original PFCP questionnaire provided the patients with an applicable tool to explore the patients experience of communication and patient-centredness throughout the encounter [35]. A third limitation is that the number of evaluation surveys per group differed. To provide transparency in the results and thereby help to visualise the students’, peers’ and clinical supervisors’ perspectives for the reader, the results were supported by citations [50].

## Implications for medical education and future research

The current study highlights that MSF, which provided feedback through the original and adapted PFCP questionnaires assessing a mutually experienced student-led patient encounter, could be a suitable learning activity to facilitate students’ and peers’ workplace learning in PHC. Furthermore, MSF could also be a suitable tool in facilitating the clinical supervisors’ pedagogical assignment during students’ workplace learning in PHC. However, further implementation studies are required to explore the aspects of this MSF learning activity as an integrated tool in students’ workplace learning. In the present study, some PHC centres only had one medical student in clinical rotation. By inclusion of students’ self-evaluation, and patients’ and clinical supervisors’ feedback, an MSF learning activity could still be accomplished as a facilitator in students’ self-directed learning regarding communication and patient-centredness. In addition to the perspective of clinical learning, it would also be interesting to explore how patients’ perspectives provided as feedback to students could influence clinical supervisors’ own clinical practice.

## Conclusion

Our findings indicate that multi-source feedback (MSF) provided directly after a patient encounter, using the original and adapted versions of the PFCP questionnaire as feedback provider, could be an adequate learning activity for medical students’ workplace learning in PHC. The MSF, provided through the PFCP questionnaire, was experienced to neutralise and operationalise the provision of concrete feedback, facilitating both students and peers’ clinical learning. Additionally, the clinical supervisors experienced that participation in the MSF learning activity added valuable patient’s perspectives to include in their clinical tuition. The results visualise the importance of patients in MSF, as a valuable resource in students’ workplace learning. Written MSF on a mutual patient encounter was found to be fairly feasible during a clinical rotation in PHC. Our study implies that this learning activity could be used as an applicable tool to facilitate learning and pedagogic development in clinical settings, such as PHC.

## Supplementary Information

**Additional file 1.** The patients’, peers’, clinical supervisors’ and students’ score, mean, (SD) and (range), for each item from the respective groups’ version of the PFCP questionnaire.

**Additional file 2.** Example of patients’, peers’, clinical supervisors’ and students’ written free-text comments in the PFCP questionnaires.

## Data Availability

The data generated and analysed during the current study are not publicly available due to ethics approval. However, data can be made available from corresponding author on reasonable request.

## Abbreviations

PHC: Primary health care
PFCP: ‘Patient Feedback in Clinical Practice’
