# Supplementary material for: Multisource feedback in medical students’ workplace learning in primary health care
Source: BMC Med Educ. 2022 May 25;22:401. doi: 10.1186/s12909-022-03468-7 (PMC9134659; doi:10.1186/s12909-022-03468-7)
Supplement: Supplementary file 1 — Additional file 1. The patients’, peers’, clinical supervisors’ and students’ score, mean, (SD) and (range), for each item from the respective groups’ version of the PFCP questionnaire. [file 12909_2022_3468_MOESM1_ESM.pdf]

Additional file 1.

File format: PDF

The participants' items' scoring in the PFCP questionnaires are presented in the Additional file 1.

	**Patient** Mean (SD) (range) (n=43)	**Peer** Mean (SD) (range) (n=20)	**Clinical supervisor** Mean (SD) (range) (n=20)	**Student** Mean (SD) (range) (n=43)
Item – 1	3.77 (0.72) (2–4)	3.95 (0.23) (3–4)	3.95 (0.22) (3–4)	3.81 (0.45) (2–4)
Item – 2	3.72 (0.70) (3–4)	3.40 (1.14) (1–4)	3.55 (1.00) (2–4)	3.44 (0.91) (1–4)
Item – 3	3.77 (0.75) (2–4)	3.05 (1.32) (2–4)	3.60 (1.05) (2–4)	3.19 (1.01) (1–4)
Item – 4	3.51 (1.20) (2–4)	3.45 (1.23) (3–4)	2.85 (1.60) (2–4)	3.35 (1.04) (2–4)
Item – 5	3.86 (0.41) (2–4)	3.65 (0.81) (1–4)	3.65 (0. 99) (2–4)	3.26 (0.95) (2–4)
Item – 6	3.47 (1.10) (2–4)	3.30 (1.03) (2–4)	3.25 (0.97) (2–4)	2.81 (1.12) (1–4)
Item – 7	3.58 (0.98) (1–4)	3.60 (0.68) (2–4)	3.00 (1.49) (1–4)	3.14 (1.10) (2–4)
Item – 8	3.81 (0.66) (3–4)	3.10 (1.55) (1–4)	3.35 (1.14) (2–4)	3.14 (1.39) (1–4)
Item – 9	3.56 (1.08) (3–4)	3.25 (1.45) (2–4)	3.25 (1.33) (1–4)	3.49 (0.91) (3–4)
Item – 10	2.86 (1.81) (3–4) (n=22)	3.50 (4–4) (1.41) (n=8)	3.50 (1.07) (1–4) (n=8)	3.00 (1.55) (3–4) (n=16)
Item – 11	2.07 (1.93) (1–4)	1.90 (2.00) (2–4)	2.00 (1.97) (1–4)	1.70 (1.88) (3–4)
Item – 12 Patient	2.50 (1.94) (3–4) (n=30)	x	x	x
Item – 13	2.35 (1.81) (2–4)	1.45 (1.73) (2–4)	1.95 (1.79) (1–4)	1.28 (1.50) (1–4)
Item – 14	4.00 (0.00) (4–4)	3.21 (1.51) (2–4) (n=19)	3.60 (0.94) (1–4)	3.21 (1.06) (3–4)
Item – 15	3.79 (0.68) (3–4)	3.35 (1.46) (3–4)	3.50 (1.05) (2–4)	3.58 (0.70) (2–4)
Item – 16	3.44 (1.36) (3–4) (n=16)	2.90 (1.62) (2–4)	3.60 (0.75) (1–4)	3.02 (1.39) (3–4) (n=42)
Item – 17	3.77 (0.81) (1–4)	2.68 (1.56) (2–4) (n=19)	3.26 (1.37) (1–4) (n=19)	2.95 (1.32) (3–4) (n=42)
Item – 18 Patient	3.81 (0.70) (2–4)	x	x	x
Item – 19	3.91 (0.29) (3–4)	4.00 (0.00) (4–4)	3.70 (0.92) (3–4)	3.77 (0.68) (1–4)
Item – 20	4.00 (0.00) (4–4)	4.00 (0.00) (4–4)	4.00 (0.00) (4–4)	3.93 (0.03) (3–4)
Item – 21 Patient	3.40 (1.30) (1–4) (n=30)	x	x	x
Item – 22 Patient	3.37 (1.22) (1–4) (n=30)	x	x	x

Additional file 1. The patients', peers', clinical supervisors' and students' score, mean, (SD) and (range), for each item from the respective groups' version of the PFCP questionnaire.
