# Supplementary material for: Multisource feedback in medical students’ workplace learning in primary health care
Source: BMC Med Educ. 2022 May 25;22:401. doi: 10.1186/s12909-022-03468-7 (PMC9134659; doi:10.1186/s12909-022-03468-7)
Supplement: Supplementary file 2 — Additional file 2. Example of patients’, peers’, clinical supervisors’ and students’ written free-text comments in the PFCP questionnaires. [file 12909_2022_3468_MOESM2_ESM.pdf]

Additional file 2.

File format: PDF

Example participants written free-texts comments from the PFCP questionnaires are presented in the Additional file 2.

Additional file 2. Example of patients', peers', clinical supervisors' and students' written free-text comments in the PFCP questionnaires.

Clinical performance	
Patients	• '. . . explained carefully what she was examining'.<br>• 'A very warm, genuine and professional demeanour during the encounter'.
Peers	• 'Good explanations'.
Clinical supervisors	• 'Summaries were partly applied'.<br>• '. . . the patient was allowed to tell his/her cause of concern. Open-ended questions and active listened were applied'.
Students	• 'The medical questions I asked were perceived as relevant'.
Suggestions for development of clinical performance	
Patients	• '…did not explain what I should do if it [my problem] would get worse (did not say what I should do then)'.
Peers	• 'A summary might have helped [the student] to obtain the patient's cause of concern'.<br>• 'He [the student] had probably not thought much about it . . . the question did not come up'.
Clinical supervisors	• '[The student] should have confirmed [verbally or non-verbally] the patient's description of previous negative experience in health care'.<br>• 'The student could increase the voice volume and decrease the conversation speed, due to the patient's slight hearing impairment'.
Students	• 'Always be sure to ask if it is ok [to ask questions or perform clinical examinations]'.<br>• 'I should provide more 'space' [for the patients in the dialogue] to ask questions'.<br>• 'The patient brought up other concerns that worried him. [I] should have elaborated them more'.
